# Cases of Fever

**Published:** 1847-04

**Authors:** John Mitchell

**Affiliations:** Jonesville, W. T.


ART. VI.—*Cases of Fever.*—By JOHN MITCHELL, M. D.

JONESVILLE, W. T., Feb. 1st. 1847.

Dr. FLINT—Dear Sir.—I notice in the Jan. number of your Journal, that Prof. Bartlett, of Lexington, Ky. is about to publish a second edition of his work on Fever and is soliciting through you, facts relating to the subject as they exist in different localities. Your invitation however is confined to N. Y. Still for "Auld acquaintancce," I flatter myself a communication from me, so far at least as you are concerned, will be acceptable.

The immortal Rush said "disease was a unit," and although I heard a few years since, a distinguished Prof. say he would have approximated nearer the truth by saying fever was a unit—yet I am inclined to think that in those days when Neuralgic and Spinal affections were little known Rush did not come far from the mark, as there are few other exceptions wherein fever is not present either idiopathic or symptomatic.

Fever is a momentous subject, and one which all medical men, in every age of the science, have been solicitous to enquire into,—and when I think of this and of how much has been written upon it, I am induced to faulter in my attempt to publish even a few observations. Indeed, I am not certain but there are many writers now, as well as formerly, who would have conferred a greater benefit upon the profession by their silence. Dr. Bartlett, as able a man as he doubtless is, may come short of his expectations in his goodly effort, if he goes beyond his own observations, and well attested facts. Medical men, doubtless, generally agree pretty well in practice; there are frequently exceptions to this however, for instance, it is not very uncommon here to administer at a dose grs. xl. and even grs. lx. of calomel, and this enormous amount is sometimes repeated. I was informed when I came here, three years ago, that this was the practice, and that I could not succeed unless I followed suit. I had no hesitation however in acting differently, and each year's experience confirms my disagreement. One morning in my rounds, during the sickly season, a friend addressed me by saying "Dr.——— of ——— or you must be wrong; he bleeds in almost every case. I happened at a house where he was attending, where there lay seven sick of fever, he was then bleeding the fifth, and was going through with the whole." Now my dear friend can you reconcile all this with good practice? With such practice as you advised in your interesting article on blood letting? If there is all this discrepancy in practice, and still more in written observations, what would a book be worth compiled from different methods practiced under like circumstances?

Our fevers here are principally Remittent and Intermittent.—They, as usual, run into each other, and put on a variety of forms. I had one case of well marked yellow fever which proved fatal.

The most malignant remittents usually run into continued Typhoid of various grades and varieties. They are usually attended with derangement of the bowels, mostly diarrhœa and distension of the abdomen—but these are by no means always present. This diarrhœa, however, is generally bilious and dependent more perhaps upon organic or functional derangement of the liver than follicular enteritis. I shall let this discription suffice for this variety of Typhoid fever, and relate a few cases of the others under this head.

Case 1. Mary D.———, an uncommonly interesting child of about one year old, in one of our principal families, the family being intimately associated with my own; and as I had attended the family several years, during which time one other child of about the same age had been lost, and attended others of the family through many serious diseases, it will readily be conceived that this case was one of no ordinary interest.

On the 27th of Oct. I found the patient labouring under fever which presented slight remissions, and diarrhœa, consisting of copious and frequent discharges. The case did not present a very alarming appearance; the diarrhœa, though obstinate, being inclined to yield. With this slight exception, she remained without any material change for three days, when the case became more alarming—diarrhœa more obstinate—fever assumed a continued form—bloating of the bowels—anxiety and other signs of distress—the symptoms daily became more agravated—she gradually sunk and died on the ninth day.

Pathological appearances. The mucus membrane of the rectum highly engorged, and many portions throughout the digestive tube injected. The glands of Peyer and Brunner generally tumefied.—In the cæcum extensive ulcerations extending from the appendix vermiformis six or eight inches, being from one to five lines in diameter. Several of the mesenteric glands much enlarged and red, and one of a dark brown and gangrenous.

Case 2d, and 3d. Father, aet. about 50, and son, 23. They were taken down simultaneously, and had been under the charge of another, and one of our best physicians some two weeks when I was called. I found the father very low—continued, low grade of fever, stupor so great that he was aroused with the greatest difficulty—muttering delirium—slight diarrhœa—tongue clean, dark, red, dry and cracked. When once aroused was perfectly rational; no pain. The son had much the same grade of fever—stupor, but in less degree—exceedingly obstinate constipation; other symptoms much the same, save that the father was disposed to sleep all the time, and the son not at all. Sordes of gums and lips in both cases.

Case 4th.—A married lady of 40, in September last had a severe course of Remittent fever, which resulted in a well marked Typhoid Pneumonia, when she became very low, but without much loss of flesh, at times had turns of sinking that required the most powerful stimulants, and tonics and in large quantities to keep soul and body together. In this way she remained without much improvement for several days, when the disease became manageable. The case now became doubly interesting. But a short time had elapsed since I looked upon it as hopeless, and now was enabled to encourage my patient with assurances of recovery ; and by diligent attention day and night, I soon had the gratification to find her sitting in her easy chair, and able to walk with assistance across the room. She gradually improved, but not without interruptions,as a matter of course, in such cases. She soon became impatient, as is usually the case in convalescence, and persisted in resorting to quack medicines, and I, of course, not being disposed nor at liberty to sanction this, was compelled to give the ground.

This patient in spite of empiricism is still living, but not entirely recovered ; and as a matter of course the credit, by the family, at least, is bestowed upon quack nostrums.

I have related this case not only to make up the variety, but to show the indefinite period such cases may run. Allowance will, of course be made for quackery, she being thrown mainly upon the resources of nature, and her constitution, and becoming very much emaciated, &c.

In this selection of cases, I have endeavored to confine myself to fevers of a typhoid character of different varieties. I am aware they will not all be regarded as French Typhoid fever. Still they are all more or less typhoid, and are dependent upon excessive irritation, or inflamation in some part of the organism. I am no great stickler, however, for technical nosology. To get at the meaning of a writer's favorite term without too much trouble, and know the disease when I see it, are sufficient for my purpose.

I could wish, that the whole nosological category was revised and abridged, and all the synonyms abolished.

This, however, I will leave, with due deference, to the champions of the science.

